# Surveillance of the Initiation of, Participation in, and Completion of Cardiac Rehabilitation in Minnesota, 2017–2018

**DOI:** 10.5888/pcd20.220324

**Published:** 2023-04-13

**Authors:** James M. Peacock, Emily Styles, Sara Johnson, Dylan Galos, Mateo Frumholtz, Shawn Leth, Aaron Pergolski

**Affiliations:** 1 Cardiovascular Health Unit, Health Promotion and Chronic Disease Division, Minnesota Department of Health, St Paul, Minnesota; 2 Office of Statewide Health Improvement Initiatives, Minnesota Department of Health, St Paul, Minnesota; 3 Infectious Disease Epidemiology, Prevention and Control Division, Minnesota Department of Health, St Paul, Minnesota; 4 Department of Cardiovascular Diseases, Mayo Clinic, Rochester, Minnesota; 5 M Health Fairview Rehab Services, Minneapolis, Minnesota

## Abstract

**Introduction:**

Cardiac rehabilitation (CR) is an evidence-based secondary prevention program designed to improve cardiovascular health after a cardiac event. The objective of our study was to identify gaps in CR use among publicly and privately insured people in Minnesota to assist in developing shared goals among public health, cardiac rehabilitation professionals, and program delivery sites to improve CR delivery.

**Methods:**

We applied a published claims-based surveillance methodology to the Minnesota All Payer Claims Database to assess eligibility for, initiation of, participation in, and completion of CR by patients with qualifying events in 2017. We stratified results by sociodemographic and geographic factors and qualifying condition and used adjusted prevalence ratios to make statistical comparisons.

**Results:**

Less than half (47.6%) of qualifying patients initiated CR within 1 year of their qualifying event; the rate was higher among men (vs women), adults aged 45 to 64 years (vs ≥65 y), and patients with commercial or Medicaid insurance coverage (vs Medicare). Among those who initiated CR, only 14.0% completed the full series of 36 sessions. Participation in at least 12 sessions and completion of 36 sessions was less likely among adults aged 18 to 64 (vs 65–74 y) and among patients covered by Medicaid (vs Medicare). Patterns of CR initiation, participation, and completion also varied geographically.

**Conclusion:**

This analysis expands on previous Medicare fee-for-service population CR surveillance and provides a first detailed look at the CR landscape in Minnesota, renewing attention to CR as a key secondary prevention strategy. Collaboration and sharing with partners has established the Minnesota Department of Health as a valuable partner in driving health system change to improve equitable provision of CR in Minnesota.

**Summary**

**What is already known on this topic?**

Previously published studies indicate that cardiac rehabilitation is an underused secondary prevention program, even though evidence demonstrates important health benefits after cardiovascular events or procedures.

**What is added by this report?**

We used published surveillance methodologies and the Minnesota All Payer Claims Database to describe the eligibility, initiation, participation, and completion of cardiac rehabilitation in Minnesota for a population of publicly and privately insured people.

**What are the implications for public health practice?**

This work demonstrated the value of all-payer claims databases to strengthen public health and health system partnerships to expand access to and provision of cardiac rehabilitation.

## Introduction

Heart disease is the leading cause of death in the US and a substantial contributor to death among middle-aged and older people. Cardiac rehabilitation (CR) is an evidence-based secondary prevention program designed to improve cardiovascular health after a person experiences a myocardial infarction, other selected cardiovascular events, or selected cardiac procedures. In outpatient CR, patients are assigned up to 36 medically supervised sessions customized to address each patient’s physical fitness, nutrition, and medication use, as well as enhance chronic disease self-management and provide psychosocial support. The few studies assessing overall CR participation have been limited to the US Medicare population (1,2) or assessed a small number of referral and enrollment metrics across multiple payers in a consortium of hospitals and health care providers in Michigan (3–6).

The primary objective of this study was to apply a standardized, published surveillance methodology for CR use to the Minnesota All Payer Claims Database (MN APCD), which includes both public and private insurers. The analysis aimed to highlight gaps in the initiation of, participation in, and completion of CR among adults in Minnesota by age, sex, geographic location, payer, qualifying condition, and other factors. Our study can inform the development of shared goals to improve access to, initiation of, participation in, and completion of CR in partnership with public health, professional organizations, and health care providers.

## Methods

We used data from the MN APCD, a large repository of health insurance claims, enrollment information, and costs for services provided to Minnesota residents (7). Both private and public insurers of Minnesota residents submit information on medical transactions for people with health insurance coverage. The database does not include claims from Tricare, Veterans Affairs, the Indian Health Service, or carriers with less than $3 million in annual medical claims or $300,000 in annual pharmacy claims. Our analysis used the 23rd extract of the MN APCD and covered the years 2017 and 2018. During this period, the MN APCD included claims for more than 95% of Minnesota’s Medicaid and Medicare enrollees and approximately 40% of commercially insured people. These deidentified data permit assessments of care delivered to patients over time and across the spectrum of the health care system (including providers, settings, and payers). The MN APCD is updated regularly and currently contains data from 2009 through 2021.

The identification of patients with qualifying conditions eligible for CR, as well as the provision of those services, followed a published surveillance methodology designed by the Million Hearts collaborative and first applied to the Medicare fee-for-service population in 2016 and 2017 (1,8). Eligible patients were identified by the presence of *International Classification of Diseases, Tenth Revision, Clinical Modification* (ICD-10-CM) diagnosis or procedure codes or Current Procedural Terminology (CPT) codes in their medical claims records (8). Primary qualifying events included acute myocardial infarction; coronary artery bypass graft surgery; heart valve repair or replacement; percutaneous coronary intervention, including percutaneous transluminal coronary angioplasty or coronary stenting; and heart or heart–lung transplant. Secondary qualifying conditions included current stable angina pectoris and stable chronic heart failure. We conducted analyses separately for patients with primary and secondary qualifying conditions. Patients were included in this analysis after having a CR-qualifying condition during 2017 and continuous health plan enrollment for 12 months after the qualifying event. Consistent with the Million Hearts CR surveillance methodology, we excluded patients in skilled nursing or hospice care, patients with end-stage renal disease, and patients without 12 months of continuous insurance enrollment after the qualifying event (1,8). Although we were not able to identify patients who died through the claims database, we excluded these patients because they did not have 12 months of continuous enrollment after the qualifying event. We assessed the frequency and timing of CR sessions per patient during the 1-year period after the qualifying event.

We applied the published surveillance methodology to a dataset of privately and publicly insured people in Minnesota, expanding on populations in previously published analyses. The MN APCD includes more than 95% of Medicare beneficiaries (fee-for-service and managed care Medicare), enrollees in Minnesota Health Care Programs (including Medical Assistance [Minnesota’s Medicaid program], and MinnesotaCare [Minnesota’s Basic Health Program]; referred to collectively as “Medicaid” hereinafter), and a large proportion (~40%) of the state’s commercially insured population.

We reported CR surveillance metrics for patients qualifying for CR in 2017 across 3 domains: initiation (percentage of patients who received CR within 1 year of a qualifying event and percentage who initiated CR within 21 days), participation (number of CR sessions within 36 weeks and percentage of patients who attended ≥12 sessions within 36 weeks); and completion (percentage of patients who attended ≥36 sessions within 36 weeks). We stratified results by sex, age group, primary payer, presence of comorbidities, 3-digit zip code area, urban–rural status, community demographics, and qualifying event. Estimates were suppressed if the population group consisted of fewer than 30 people in the denominator (eligible patients) or fewer than 11 people in the numerator (patients meeting the CR indicator) of any of the surveillance metrics, following MN APCD suppression guidelines. The MN APCD currently does not include information on race or ethnicity, despite being collected by Medicare, Medicaid, and other payers.

For payer stratifications, patients were attributed to the plan in which they were enrolled during the month of their qualifying event. Patients were not excluded from analysis if they changed payer during the follow-up period (for example, switching from commercial health insurance to Medicare) as long as they were represented in the data set for 12 months continuously. This method contrasts with the methods of national surveillance reports, which include only patients with continuous enrollment in a single health plan (eg, Medicare fee-for-service) for the entire follow-up period.

The presence of comorbid conditions was identified by using the Johns Hopkins ACG System version 12.0. The ACG system is a population health analytics software that includes a tool to create patient markers for selected high-prevalence chronic conditions based on diagnosis and pharmacy information (9). We selected conditions from the ACG system that were largely aligned with conditions described by Ritchey et al (1), including vision problems (macular degeneration and glaucoma), mental illness (bipolar, depression, and schizophrenia), congestive heart failure, endocrine/renal dysfunction (diabetes, hypothyroidism, and chronic renal failure), musculoskeletal and neurologic disorders (osteoporosis, rheumatoid arthritis, Parkinson disease, and seizure disorders), respiratory diseases (persistent asthma and chronic obstructive pulmonary disease), and currently receiving cancer treatment.

The MN APCD provides data on patient residence at the 5-digit zip code level. To assess differences by geographic regions of Minnesota, we assigned patients to 15 distinct geographies based on the first 3 digits of their home zip code (2 adjacent regions were combined because of small populations, 556 and 557). We used zip code tabulation areas to approximate the geographic boundaries of these regions (10). We used Esri ArcGIS Desktop/ArcMap version 10.8 to create maps that show the variation in 4 CR outcomes by 3-digit zip code regions and the location of outpatient CR sites. Seventy-nine of 87 counties have at least 1 outpatient CR program (11). We used quartiles to describe the distribution of patients by CR metrics; the first quartile includes the 25% of zip code regions with the lowest values. 

We used 2010 rural–urban commuting area (RUCA) codes (12) to stratify zip code data and assess differences among urban and rural communities. In decreasing order of density, these communities were metropolitan (living in or commuting to an urban area of ≥50,000 population), micropolitan (urban area population of 10,000–49,999), small town (urban area population of 2,500–9,999), and rural (no urban population). To assess any differences by place-based sociodemographic characteristics, we used the Centers for Disease Control and Prevention (CDC)/Agency for Toxic Substances and Disease Registry’s Social Vulnerability Index (SVI), which classifies census tracts into levels of social vulnerability based on 15 factors related to socioeconomic status, household composition, race, ethnicity, language, and housing/transportation (13). As part of our agency’s coordinated COVID-19 response, the Minnesota Department of Health created a zip code–based SVI index to track COVID-19 trends and to inform vaccine prioritization beginning in 2021 (14). SVI scores were crosswalked from census tracts to zip codes by using data sets provided by the US Department of Housing and Urban Development (15) and following established methods (16,17).

Finally, we tabulated CR outcomes by primary qualifying conditions, including with and without procedures (for events) and with and without events (for procedures), and by secondary qualifying conditions.

To compare stratified groups, we calculated adjusted prevalence ratios (aPRs), adjusted for age, sex, and payer, as appropriate. Unadjusted prevalence estimates are presented alongside aPRs. We used Proc Genmod (SAS Enterprise Guide version 7.1), a Poisson distribution, and a log link function to conduct analyses. 

## Results

During 2017, the MN APCD included data on 3,806,842 members, approximately 69% of Minnesota’s population, and we identified 19,974 adults with a primary qualifying event for CR. After we applied exclusion criteria, 12,937 members in the MN APCD with a primary qualifying event for CR were eligible for analysis (Table 1); they collectively engaged in 118,475 billable CR sessions within 1 year after their qualifying event. The group had approximately twice as many men (n = 8,740) as women (n = 4,197), and 71.5% (n = 9,246) had Medicare as the primary payer; the remainder were insured by commercial plans, Medicaid, or were dually insured by Medicare and Medicaid. These data underestimate the total eligible population in our state, because we did not have claims from approximately 60% of commercial beneficiaries, patients using other federal health care payment mechanisms, and a small proportion who were uninsured.

**Table 1:** Cardiac Rehabilitation Eligibility, Initiation, Participation, and Completion Among Adults Aged ≥18 Years, Minnesota, 2017–2018

Characteristic	No. of patients qualifying for CR^a^	Patients with any CR within 1 year		Patients initiating CR within 21 days		No. of CR sessions within 36 weeks, mean (SD)	Patients with ≥12 sessions within 36 weeks		Patients with ≥36 sessions within 36 weeks	
		No. (%)	aPR^b^ (95% CI)	%	aPR^b^ (95% CI)		%	aPR^b^ (95% CI)	%	aPR^b^ (95% CI)
**All**	12,937	6,154 (47.6)	—	69.8	—	19.0 (13.1)	65.5	—	14.0	—
**Sex**										
Female	4,197	1,798 (42.8)	0.90 (0.85–0.95)^c^	66.4	0.94 (0.88–1.01)	18.7 (13.4)	63.7	0.96 (0.90–1.03)	13.2	0.91 (0.78–1.05)
Male	8,740	4,356 (49.8)	Reference	71.2	Reference	19.2 (12.9)	66.3	Reference	14.4	Reference
**Age group, y**										
18–44	382	201 (52.6)	1.05 (0.91–1.22)	66.7	0.95 (0.80–1.14)	12.9 (12.3)	44.3	0.61 (0.50–0.76)^c^	6.0	0.39 (0.22–0.69)^c^
45–64	3,341	1,936 (58.0)	1.15 (1.09–1.23)^d^	72.3	1.02 (0.95–1.10)	17.1 (12.9)	58.2	0.81 (0.75–0.87)^c^	11.8	0.76 (0.64–0.89)^c^
65–74	4,576	2,295 (50.2)	Reference	70.5	Reference	20.8 (13.2)	71.8	Reference	15.6	Reference
75–84	3,387	1,402 (41.4)	0.83 (0.78–0.89)^c^	66.6	0.95 (0.87–1.03)	20.1 (12.3)	69.9	0.98 (0.90–1.06)	16.3	1.05 (0.89–1.24)
≥85	1,251	320 (25.6)	0.52 (0.46–0.58)^c^	65.6	0.94 (0.81–1.08)	17.5 (13.4)	58.8	0.82 (0.71–0.96)^c^	11.9	0.77 (0.55–1.08)
**Type of health insurance**										
Commercial	2,242	1,545 (68.9)	1.50 (1.39–1.61)^d^	77.7	1.15 (1.05–1.25)^d^	19.7 (12.9)	68.2	0.97 (0.89–1.06)	15.7	1.30 (1.08–1.58)^d^
Dual Medicare and Medicaid	194	59 (30.4)	0.67 (0.52–0.87)^c^	64.4	0.95 (0.68–1.32)	17.5 (12.1)	62.7	0.89 (0.64–1.24)	6.4^e^	0.58 (0.41–0.80)^c^^,^ e
Medicaid	1,246	686 (55.1)	1.18 (1.07–1.30)^d^	64.4	0.96 (0.84–1.08)	11.4 (11.8)	34.3	0.49 (0.42–0.57)^c^		
Medicare	9,246	3,858 (41.7)	Reference	67.7	Reference	20.2 (12.9)	70.1	Reference	14.9	Reference
**No. of comorbidities^f^**										
0 or 1	6,266	3,338 (53.3)	Reference	74.7	Reference	19.0 (12.7)	66.3	Reference	13.9	Reference
2 or 3	5,464	2,377 (43.5)	0.88 (0.84–0.93)^c^	65.2	0.89 (0.83–0.95)^c^	19.2 (13.3)	65.5	1.00 (0.94–1.07)	14.1	1.02 (0.88–1.18)
≥4	1,207	439 (36.4)	0.78 (0.70–0.86)^c^	57.9	0.80 (0.70–0.91)^c^	18.8 (14.5)	59.5	0.91 (0.80–1.03)	14.7	1.08 (0.83–1.41)
**Mental illness**										
No	9,510	4,595 (48.3)	Reference	71.3	Reference	19.4 (13.0)	67.3	Reference	14.6	Reference
Yes	3,427	1,559 (45.5)	0.94 (0.88–1.00)	65.5	0.93 (0.87–1.00)	17.9 (13.3)	60.2	0.95 (0.88–1.02)	12.3	0.94 (0.80–1.11)

Abbreviations: —, does not apply; aPR, adjusted prevalence ratio; CR, cardiac rehabilitation.

a Totals for stratified groups will not sum to the total analysis population if information was missing.

b Statistical comparisons between groups are evaluated by using prevalence ratios (α = .05) adjusted for age, sex, and payer, as appropriate.

c Significantly lower than the reference group.

d Significantly higher than the reference group.

e Groups were combined for this indicator and cells were merged because individual results for the dual eligible population did not meet suppression thresholds defined by the data set administrator.

f Includes vision problems, mental illness, congestive heart failure, endocrine/renal dysfunction, musculoskeletal disorders, respiratory diseases, and cancer treatment.

Among qualifying patients, 47.6% initiated CR within 1 year after a qualifying event (Table 1). Of participating patients, 69.8% initiated their first session within 21 days of their qualifying event. Participating patients averaged 19.0 sessions; 65.5% completed 12 or more sessions, and 14.0% completed 36 sessions or more within 36 weeks. After adjusting for age and insurance type, we found that women were less likely than men to initiate CR (42.8% vs 49.8%; aPR, 0.90 [95% CI, 0.85–0.95]). Of the 5 age groups, compared with adults aged 65 to 74 years, adults aged 45 to 64 years were most likely to initiate CR (58.0%; aPR, 1.15 [95% CI, 1.09–1.23]), while participation rates declined among older adults (41.4% among those aged 75–84 years [aPR, 0.83; 95% CI, 0.78–0.89] and 25.6% among those aged ≥85 years [aPR, 0.52 [95% CI, 0.46–0.58]).

Medicare beneficiaries represented 71.5% of the primary qualifying population, and 41.7% participated in CR within 1 year after their primary event. After adjusting for age, compared with the Medicare population, both the commercially insured (aPR, 1.50; 95% CI, 1.39–1.61) and Medicaid (aPR, 1.18; 95% CI, 1.07–1.30) populations were more likely to participate, while the dual Medicare and Medicaid (aPR, 0.67; 95% CI, 0.52–0.87) population was less likely. However, after initiation of CR, only the commercially insured population was more likely than the Medicare population (aPR, 1.30; 95% CI, 1.08–1.58) to complete 36 sessions or more within 36 weeks, while the dual Medicare and Medicaid population was less likely (aPR, 0.58; 95% CI, 0.41–0.80). In general, patients with 2 or more comorbid conditions were less likely than patients with 0 or 1 comorbidities to participate and were slower to initiate CR, but we found no differences in completion of sessions. We found no significant differences in CR outcomes when assessing mental illness as a comorbidity.

In our examination of CR outcomes by RUCA codes, the only significant difference was a higher likelihood of patients completing 36 or more CR sessions within 36 weeks in micropolitan and small town communities compared with metropolitan areas (Table 2). As expected, we observed a larger number of CR-qualifying patients in more socially vulnerable communities than in less socially vulnerable communities. By social vulnerability, the only significant difference in CR outcomes was a higher prevalence of patients completing 36 or more CR sessions in the second most vulnerable quartile (quartile 2) compared with the least vulnerable quartile (quartile 4).

**Table 2:** Geographic Variation in Cardiac Rehabilitation Eligibility, Initiation, Participation, and Completion Among Adults Aged ≥18 Years, Minnesota, 2017–2018

Characteristic	No. of patients qualifying for CR^a^	Patients with any CR within 1 year		Patients initiating CR within 21 days		No. of CR sessions within 36 weeks, mean (SD)	Patients with ≥12 sessions within 36 weeks		Patients with ≥36 sessions within 36 weeks	
		No. (%)	aPR^b^ (95% CI)	%	aPR^b^ (95% CI)		%	aPR^b^ (95% CI)	%	aPR^b^ (95% CI)
**All**	12,937	6,154 (47.6)	—	69.8	—	19.0 (13.1)	65.5	—	14.0	—
**Rural–urban commuting area code^c^**										
Metropolitan	7,841	3,773 (48.1)	Reference	71.2	Reference	18.9 (12.6)	65.6	Reference	13.3	Reference
Micropolitan	1,962	939 (47.9)	1.06 (0.99–1.14)	68.2	0.98 (0.89–1.06)	20.7 (14.6)	69.9	1.07 (0.98–1.17)	17.3	1.31 (1.09–1.56)^d^
Small town	1,306	621 (47.6)	1.06 (0.97–1.15)	64.7	0.93 (0.84–1.03)	19.2 (12.7)	64.3	0.99 (0.89–1.10)	16.7	1.29 (1.05–1.60)^d^
Rural	1,789	803 (44.9)	0.99 (0.92–1.07)	69.2	0.99 (0.90–1.09)	17.8 (13.2)	61.3	0.93 (0.85–1.03)	12.0	0.91 (0.73–1.13)
**Social Vulnerability Index^e^ quartiles**										
Quartile 1 (most vulnerable)	4,354	2,010 (46.2)	0.94 (0.88–1.02)	68.1	0.95 (0.88–1.04)	17.9 (12.8)	60.5	0.92 (0.84–1.00)	14.0	1.21 (1.00–1.48)
Quartile 2	3,205	1,483 (46.3)	0.96 (0.89–1.03)	69.5	0.97 (0.89–1.06)	19.9 (13.8)	67.4	1.00 (0.91–1.09)	16.3	1.35 (1.10–1.66)^d^
Quartile 3	2,841	1,385 (48.8)	0.99 (0.92–1.07)	69.5	0.96 (0.88–1.05)	19.4 (12.9)	67.5	0.98 (0.89–1.07)	13.2	1.07 (0.86–1.32)
Quartile 4 (least vulnerable)	2,468	1,247 (50.5)	Reference	73.1	Reference	19.6 (12.7)	69.6	Reference	12.4	Reference
**3-Digit zip code area^f^**										
550 (Twin Cities East)	1,642	819 (49.9)	1.11 (1.00–1.22)	68.4	0.93 (0.83–1.05)	20.0 (13.3)	66.9	0.97 (0.87–1.09)	15.4	1.26 (0.97–1.48)
551 (St. Paul)	1,657	828 (50.0)	1.10 (1.00–1.21)	67.8	0.93 (0.83–1.04)	17.0 (11.7)	61.0	0.91 (0.81–1.02)	10.5	0.89 (0.67–1.18)
553 (Twin Cities West)	1,926	938 (48.7)	1.08 (0.98–1.18)	74.1	1.01 (0.91–1.12)	18.9 (11.9)	69.1	1.00 (0.89–1.12)	9.4	0.77 (0.58–1.02)
554 (Minneapolis)	1,888	853 (45.2)	Reference	73.0	Reference	19.2 (12.6)	66.9	Reference	11.8	Reference
556/557 (Hibbing)	658	322 (48.9)	1.15 (1.01–1.30)^d^	66.1	0.93 (0.79–1.08)	20.3 (14.2)	62.4	0.95 (0.80–1.11)	22.4	1.93 (1.42–2.61)^d^
558 (Duluth)	289	134 (46.4)	1.03 (0.86–1.24)	79.1	1.08 (0.88–1.33)	21.3 (14.4)	64.9	0.99 (0.79–1.24)	25.4	2.19 (1.49–3.23)^d^
559 (Rochester)	789	391 (49.6)	1.07 (0.95–1.20)	73.9	1.00 (0.87–1.14)	22.4 (13.8)	69.3	1.02 (0.89–1.18)	29.9	2.40 (1.84–3.14)^d^
560 (Mankato)	672	316 (47.0)	1.10 (0.97–1.26)	68.7	0.95 (0.81–1.11)	21.2 (12.6)	74.4	1.07 (0.92–1.25)	15.5	1.23 (0.87–1.74)
561 (Worthington)	360	127 (35.3)	0.83 (0.69–1.00)	70.1	0.97 (0.78–1.21)	19.1 (11.9)	66.1	1.00 (0.80–1.26)	11.8	1.01 (0.59–1.73)
562 (Willmar)	369	170 (46.1)	1.08 (0.92–1.27)	70.0	0.97 (0.80–1.18)	21.7 (13.2)	72.9	1.08 (0.88–1.31)	21.2	1.73 (1.18–2.54)^d^
563 (St. Cloud)	814	404 (49.6)	1.13 (1.00–1.27)	59.9	0.82 (0.71–0.95)^g^	17.3 (12.5)	65.1	0.95 (0.82–1.10)	7.9	0.65 (0.44–0.97)^g^
564 (Brainerd)	586	269 (45.9)	1.07 (0.93–1.22)	61.0	0.85 (0.71–1.01)	15.2 (11.5)	52.8	0.79 (0.65–0.95)^g^	8.2	0.69 (0.44–1.10)
565 (Moorhead)	596	277 (46.5)	1.11 (0.97–1.27)	75.1	1.06 (0.90–1.24)	22.6 (18.5)	69.3	1.02 (0.86–1.20)	23.5	1.95 (1.2–2.67)^d^
566 (Bemidji)	487	226 (46.4)	1.09 (0.94–1.26)	73.0	1.03 (0.86–1.22)	12.6 (10.0)	49.6	0.76 (0.62–0.93)^g^	6.7^h^	0.59 (0.37–0.96)^g^^,^ h
567 (Thief River Falls)	189	72 (38.1)	0.90 (0.71–1.14)	55.6	0.78 (0.56–1.07)	18.4 (13.6)	61.1	0.93 (0.68–1.26)		

Abbreviations: —, does not apply; aPR, adjusted prevalence ratio; CR, cardiac rehabilitation.

a Totals for stratified groups will not sum to the total analysis population due to patients who were missing zip code information.

b Statistical comparisons between groups were evaluated by using prevalence ratios (α = .05) adjusted for age, sex, and payer, as appropriate.

c Rural–urban commuting area (RUCA) codes (12) were used to stratify zip code data and assess differences among urban and rural communities: metropolitan (living in or commuting to an urban area of ≥50,000 population), micropolitan (urban area population of 10,000–49,999), small town (urban area population of 2,500–9,999), and rural (no urban population).

d Significantly higher than the reference group; Minneapolis Zip Code region 554 was chosen as the reference group because performance on CR metrics in this region was most similar to that of Minnesota overall.

e Centers for Disease Control and Prevention/Agency for Toxic Substances and Disease Registry’s Social Vulnerability Index (SVI) classifies census tracts into levels of social vulnerability based on 15 factors related to socioeconomic status, household composition, race, ethnicity, language, and housing or transportation (13).

f The largest city or region in each zip code area listed to provide reference.

g Significantly lower than the reference group; Minneapolis zip code region 554 was chosen as the reference group because performance on CR metrics in this region was most similar to that of Minnesota overall.

h Adjacent zip code groups were combined and cells were merged for this indicator because individual results for group 567 did not meet suppression thresholds defined by the data set administrator.

Our maps (Figure) show that zip code region 565, in western Minnesota surrounding the city of Moorhead, had lower rates of initiation (A) but performed in the highest quartile for timely initiation (B) and completion of 12 or more and 36 or more sessions (C and D). Region 563, in central Minnesota surrounding St. Cloud, had one of the highest initiation rates but was in the lowest quartile for timely initiation and completion of 36 or more sessions. Region 559, in southeastern Minnesota surrounding Rochester, performed well across all metrics, while regions in north central (564) and northwestern (567) Minnesota had low rates overall. 

**Figure:**
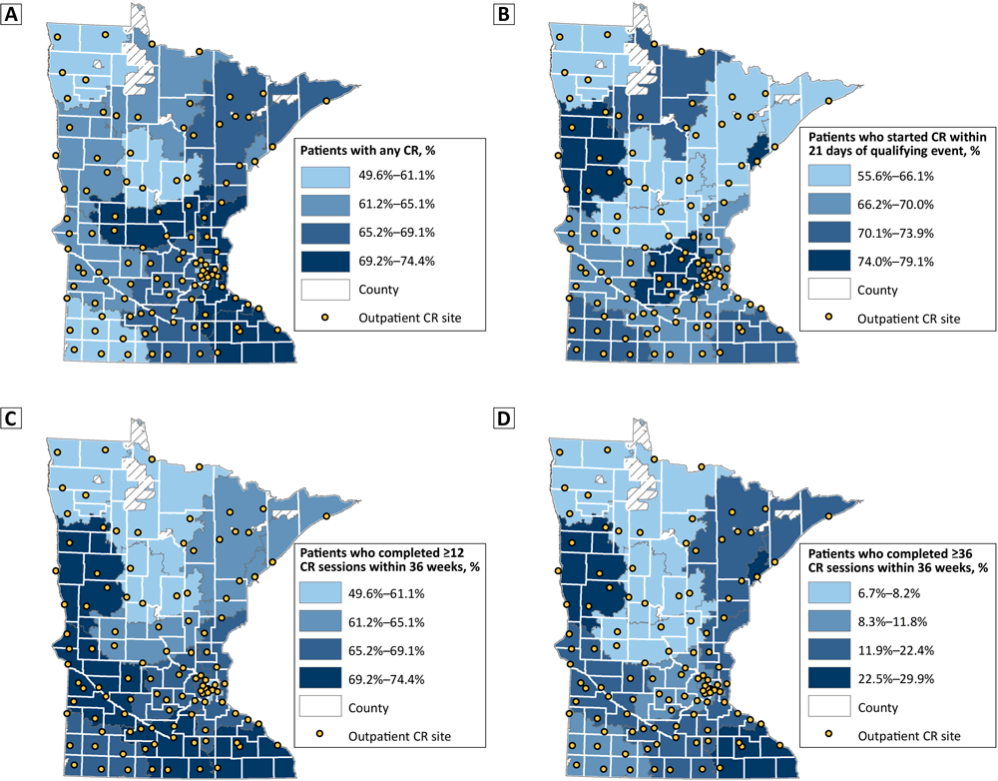
Initiation, participation, and completion of cardiac rehabilitation among adults aged ≥18 years in Minnesota, by 3-digit zip code regions, 2017–2018. Hash marks indicate areas not covered by zip codes.

The most common primary qualifying conditions were AMI and percutaneous transluminal coronary angioplasty (Table 3). Although we did not make statistical comparisons, patients with coronary artery bypass graft surgery were the most likely to participate in CR (70.2%) and the most likely to complete 36 or more sessions within 36 weeks (18.4%). Patients with AMI and no procedure were the least likely to participate in CR (16.8%).

**Table 3:** Cardiac Rehabilitation Eligibility, Initiation, Participation, and Completion Among Adults Aged ≥18 Years, by Qualifying Condition or Procedure, Minnesota, 2017–2018

Condition	No. of patients qualifying for CR^a^	No. of patients with any CR within 1 year, no. (%)	Patients initiating CR within 21 days, %	Mean no. of CR sessions within 1 year	Patients with ≥12 sessions within 36 weeks, %	Patients with ≥25 sessions within 36 weeks, %	Patients with ≥36 sessions within 36 weeks, %
**Primary qualifying conditions**							
All	12,937	6,154 (47.6)	69.8	19.0	65.5	33.2	14.0
**AMI**							
All	5,968	2,510 (42.1)	71.8	18.7	64.7	32.7	13.4
Without procedure	2,162	364 (16.8)	52.7	17.9	59.3	30.2	12.9
With procedure	3,806	2,146 (56.4)	75.0	18.9	65.6	33.1	13.5
**Coronary artery bypass graft**							
All	1,393	978 (70.2)	62.5	21.2	73.6	38.7	18.4
Without AMI	813	578 (71.1)	59.0	21.2	71.8	38.4	19.4
With AMI	349	242 (69.3)	67.4	20.5	74.0	35.1	17.8
**Percutaneous transluminal coronary angioplasty**							
All	7,837	3,980 (50.8)	73.4	18.4	62.9	31.7	12.9
Without AMI	4,358	2,068 (47.5)	71.2	18.1	61.5	30.6	12.8
With AMI	3,365	1,847 (54.9)	76.2	18.6	64.2	32.5	12.9
**Other**							
Valve repair	1,801	998 (55.4)	68.4	20.6	72.4	37.0	15.2
Transplant	33	15 (45.5)	^—b^	21.6	^—b^	^—b^	^—b^
**Multiple procedures**							
All	289	181 (62.6)	65.2	22.2	79.0	45.3	17.1
Without AMI	220	136 (61.8)	64.0	22.5	78.7	45.6	17.6
With AMI	69	45 (65.2)	68.9	22.0	80.0	44.4	^—b^
**Secondary qualifying conditions**							
All	16,337	516 (3.2)	—c	18.1	57.6	28.3	10.7
Angina	2,014	103 (5.1)	—c	21.3	67.0	32.0	15.5
Heart failure	14,323	413 (2.9)	—c	17.3	55.2	27.4	9.4

Abbreviations: AMI, acute myocardial infarction; CR, cardiac rehabilitation.

a Qualifying conditions are not mutually exclusive and totals for stratified groups will not sum to the total analysis population.

b Result did not meet suppression thresholds defined by the data set administrator.

c Indicator was not calculated for patients with secondary qualifying conditions.

Only 3.2% of patients with secondary qualifying conditions initiated CR within 1 year of a qualifying event, ranging from 5.1% of patients with chronic stable angina to 2.9% of patients with heart failure (Table 3). Mean number of CR sessions completed within 1 year and percentages of patients completing 12 or more sessions and 36 or more sessions were lower for patients with secondary qualifying conditions than for those with primary qualifying conditions.

## Discussion

This surveillance analysis is the first population-based assessment of the initiation of, participation in, and completion of outpatient CR in Minnesota using administrative claims data. It expands on previous estimates from telephone-based health surveys (18) and estimates from analyses restricted to Medicare fee-for-service data at the national, regional (1), and state level (2–6). Our analysis was catalyzed by funding from CDC’s Division for Heart Disease and Stroke Prevention and the Million Hearts initiative, which have highlighted CR as a key strategy to improve cardiovascular disease outcomes in the US (19). The Minnesota Department of Health is committed to using data to improve the state’s overall population health and working toward the ambitious Million Hearts goal of 70% participation in CR.

Our study has several limitations. The main limitation is that we were missing a sizable proportion of the commercially insured population. That proportion is missing because of the Gobeille vs Liberty Mutual Insurance Company ruling, which stated that self-insured commercial plans covered by the federal Employee Retirement Income Security Act of 1974 cannot be required to follow a state data submission requirement (20). This ruling has resulted in data gaps in APCDs. Adjusting our participation estimates to account for the missing 60% of commercial beneficiaries increased overall participation in CR from 47.6% to 52.0%, with increases across regions ranging from 2.2 to 5.7 percentage points.

Second, because the MN APCD is an administrative claims data set, it limits our ability to analyze data through a health equity lens. While we could stratify data by sex, age, payer, rural residence, and comorbid conditions, we could not stratify data by patients’ race, ethnicity, preferred language, or other sociodemographic factors because the MN APCD does not collect these data. As an alternative approach, we used the SVI of the patient’s zip code to approximate their sociodemographic characteristics. Third, the MN APCD includes information only on events and procedures that are reimbursed by insurance through a claim. Information on home-based and hybrid CR programs, while growing in popularity and sophistication, is not captured by the data set. Fourth, the surveillance methodology excluded patients who experienced short gaps in coverage, which may have under- or overestimated actual CR participation. Inconsistent coverage is especially prevalent in the Medicaid population, in which the typical beneficiary is covered for less than 10 months of the year (21). Fifth, although a complete course of CR typically includes 36 sessions, patients who meet their goals may be discharged early, which would not have been captured by our completion metric. Finally, these results describe the provision of CR in Minnesota before the COVID-19 pandemic, which has had major impacts on care patterns, referrals, and attendance at center-based CR programs. Recent analysis of Medicare data nationally showed that CR participation continued to lag prepandemic levels as recently as late 2021 (22).

Despite these limitations, these results have expanded our understanding of the CR landscape in Minnesota and have focused new attention on CR as a key secondary prevention strategy critical to optimal recovery from cardiovascular events and procedures. This work builds on our efforts to demonstrate the value of all-payer claims databases, which currently serve as the primary tool for assessing the frequency and cost of medical services provided to people in Minnesota. It also continues our efforts to use the MN APCD for public health surveillance purposes, following previous reports describing hypertension prevalence and blood pressure medication nonadherence (23,24). The MN APCD allows us to expand surveillance of CR beyond the Medicare fee-for-service population to a broader range of payers, including more than 95% of Medicare and Medicaid enrollees and approximately 40% of those in commercial plans. The proportion of Medicare beneficiaries in Minnesota who are enrolled solely in fee-for-service Medicare is among the lowest in the US, at approximately 50% (25). Because the MN APCD also includes managed-care Medicare plans, this analysis of patients with qualifying events in 2017 identified more than 2.5 times as many CR-eligible Medicare beneficiaries (9,246 vs 3,541), as previously published results among the Medicare fee-for-service population in Minnesota (2). Our analysis showed lower rates of initiation (41.7% vs 51.7%), more timely initiation (67.7% vs 36.4%), and similar rates of completion of 12 or more (74.9% vs 70.1%) or 36 or more sessions (14.9% vs 15.5%) in Minnesota’s Medicare population (2).

This work also helps to fill information gaps that exist because health systems typically have access to data only on their own patients’ use of services in their own system. The MN APCD contains claims for patients across payers and health care systems, allowing us to track service use in a way individual health systems cannot. CR often takes place in a different community or health system than where the qualifying event occurred; thus, patient care is often not limited to 1 health system or 1 location. This complex web of care creates barriers to assessing population-level CR participation. The MN APCD overcomes this challenge by assessing patient experiences across multiple care systems, inside and outside Minnesota.

Although likely to be of high value to our Minnesota partners, this article describes only a portion of the results of our analysis. Our approach could be useful in other states or jurisdictions with sufficient data or an APCD. The Minnesota Department of Health is well positioned to conduct and share this type of population-level surveillance and provide an unbiased view of CR service delivery in our state. Regional surveillance data are integral to informing program planning at state and local levels and engaging CR delivery sites. Identifying regions with higher participation and completion rates could encourage health systems in those areas to share the policies and practices that contribute to the success of their programs with other health systems in the state.

To further that end, results of these analyses are being shared with our partners statewide to encourage growth in CR services, identify gaps in CR provision, and develop goals that are both practical and aspirational. This work highlights key inequities in CR participation, notably by age, payer, geography, and qualifying condition. Addressing these inequities with new and novel solutions requires strong partnerships at multiple levels. The Minnesota Association of Cardiovascular and Pulmonary Rehabilitation, an affiliate of the American Association of Cardiovascular and Pulmonary Rehabilitation, has become a key partner; preliminary results from 2016 were presented as continuing education for more than 100 attendees at its 2021 state conference. Those results were also shared with one of the largest health systems in Minnesota to inform strategic efforts to address gaps in CR participation. Identifying opportunities for increased CR contributed to policy changes in that health system to cover parking costs for CR patients at key locations, long identified as a cost barrier for patients. In addition, these data have informed new efforts within the Minnesota Department of Health and health systems to use hospital discharge and electronic health record data to identify equity gaps in CR referral, initiation, participation, and completion. Our analysis is a catalyst for future collaboration between CR programs and public health to provide more equitable access to CR throughout Minnesota and further establishes the Minnesota Department of Health as a valuable partner in driving health system change and improved heart health.
