# Erratum, Vol. 20, April 13, 2023 Release

**DOI:** 10.5888/pcd20.220324e

**Published:** 2023-05-11

The key for map A in the figure in the article “Surveillance of the Initiation of, Participation in, and Completion of Cardiac Rehabilitation in Minnesota, 2017–2018” was incorrect as originally published. The key was corrected, and the revised figure was posted on April 24, 2023: https://www.cdc.gov/pcd/issues/2023/22_0324.htm. We regret any confusion this error may have caused.

